# CD18 Regulates Monocyte Hematopoiesis and Promotes Resistance to Experimental Schistosomiasis

**DOI:** 10.3389/fimmu.2018.01970

**Published:** 2018-08-31

**Authors:** Camila O. S. Souza, Milena S. Espíndola, Caroline Fontanari, Morgana K. B. Prado, Fabiani G. Frantz, Vanderlei Rodrigues, Luiz G. Gardinassi, Lúcia H. Faccioli

**Affiliations:** ^1^Departamento de Análises Clínicas, Toxicológicas e Bromatológicas, Faculdade de Ciências Farmacêuticas de Ribeirão Preto, Universidade de São Paulo, São Paulo, Brazil; ^2^Departamento de Bioquímica e Imunologia, Faculdade de Medicina de Ribeirão Preto, Universidade de São Paulo, São Paulo, Brazil

**Keywords:** β_2_ integrin, schistosomiasis, monocytes, hematopoiesis, immune regulation, resistance

## Abstract

Infection with *Schistosoma mansoni* causes a chronic parasitic disease that progress to severe liver and gastrointestinal damage, and eventually death. During its development into mammalian hosts, immature schistosomula transit through the lung vasculature before they reach the liver to mature into adult worms. A low grade inflammatory reaction is induced during this process. However, molecules that are required for efficient leukocyte accumulation in the lungs of *S. mansoni*-infected subjects are unknown. In addition, specific leukocyte subsets that mediate pulmonary response during *S. mansoni* migration through the lung remain to be elucidated. β_2_ integrins are fundamental regulators of leukocyte trans-endothelial migration and function. Therefore, we investigated their role during experimental schistosomiasis. Mice that express low levels of CD18 (the common β_2_ integrin subunit) and wild type C57BL/6 mice were subcutaneously infected with *S. mansoni* cercariae. Cellular profiles of lungs and livers were evaluated in different time points after infection by flow cytometry. Low levels of CD18 affected the accumulation of patrolling Ly6C^low^, intermediate Ly6C^inter^ monocytes, monocyte-derived macrophages and monocyte-derived dendritic cells in the lungs 7 days after infection. This correlated with increased TNF-α levels. Strikingly, low CD18 expression resulted in monocytopenia both in the peripheral blood and bone marrow during acute infection. After 48 days, *S. mansoni* worm burdens were higher in the hepatic portal system of CD18^low^ mice, which also displayed reduced hepatic accumulation of patrolling Ly6C^low^ and intermediate Ly6C^inter^, but not inflammatory Ly6C^high^ monocytes. Higher parasite burden resulted in increased granulomatous lesions in the liver, increased egg deposition and enhanced mortality. Overall, our data point for a fundamental role of CD18 for monocyte hematopoiesis during infection, which promotes an efficient host response against experimental schistosomiasis.

## Introduction

Schistosomiasis is a neglected helminthic disease caused by worms of the genus *Schistosoma* spp. (1). According to WHO, the disease affects millions of people in tropical and subtropical regions, and approximately 200,000 fatal outcomes per year have been estimated in the sub-Saharan Africa (2). After infective cercariae penetrate the host skin, they differentiate into endoparasitic larvae, the schistosomula. The parasites penetrate the skin within the first hours and migrate through systemic vasculature circuit, peaking in the lungs between 5 and 7 days of infection (3). Larvae that pass through the lung vasculature are delivered to the hepatoportal circulation, where they mature into adult worms and later migrate to mesenteric venules, mate, and begin egg deposition (4, 5).

During the acute phase of schistosomiasis, innate immune cells are activated and predominantly produce cytokines such as TNF-α, IL-2, IL-6, and IL-1β. When eggs are produced, this cytokine profile changes dramatically. Indeed, chronic schistosomiasis is characterized by the high levels of IL-4, IL-5, IL-13, and IL-10 which trigger type 2 granuloma responses (6, 7). The balance between cytokines during early and later disease stages can determine the clinical outcome. After infection with *S. mansoni*, IL-4 deficient mice produce higher amounts of IFN-γ and TNF-α, but develop a severe and fatal disease (8). Beyond cytokine production, specialized innate immune cells drive the activation and polarization of adaptive immune responses mediated by T and B lymphocytes. During *S. japonica* infection, monocyte-derived dendritic cells (MDCs) produce IL-4 to trigger Th2 responses in the liver (9). These cells are commonly known as inflammatory DCs, characterized by the surface expression of CD11b^+^, CD11c^+^, MHC-II^+^, CD40^+^, CD86^+^, and Ly6C^high^ (10). Distinct murine blood monocyte subsets display different molecular programs, which will favor the differentiation of MDC or monocyte-derived macrophages (MDM) (11, 12). However, the trafficking of such cells to affected tissues depends on chemokines, bioactive lipids, and molecules involved in cellular adhesion (13, 14). Ly6C^high^ monocytes give rise to alternatively activated macrophages in liver granulomas of *S. mansoni*-infected mice (15, 16), requiring the activity of CCL2/CCR2 axis (16). Seminal studies in mice lead to the important observation that lungs of *S. mansoni*-infected animals, and not the skin, promote the greatest obstacle for further parasite migration in the vasculature (3, 17). Schistosomula trapped in lung capillaries induce a low grade inflammatory response (3). Pulmonary endothelial cells (ECs) are activated after *S. mansoni* infection, increase the expression of the adhesion molecule ICAM-1 and facilitate leukocyte infiltration (18). Indeed, the lung has been proposed to function as a vascular filter and site for induction T cell responses to large blood-borne pathogens, such as helminths (19). However, the dynamics of innate immune cell responses during *S. manoni* migration through the lung and the possible implications for latter outcomes remain poorly understood.

Integrins are fundamental molecules for leukocyte adhesion and trans-endothelial migration. Their structures are formed by the non-covalent association of one α-subunit and one β-subunit. The functional β_2_ integrin subunit (CD18) partners with different α-subunits (αL—CD11a, αM—CD11b, αX—CD11c, and αD—CD11d) to form specific molecules. The interaction with different ligands triggers specific immune cell functions, such as adhesion to endothelium or even cell signaling promoted by anaphylatoxins of the complement cascade (20). CD18 is important for efficient adhesion of eosinophils and neutrophils in lung capillaries, and they are required to maintain macrophage effector functions after stimulus with protein extracts or eggs of *S. mansoni* (21, 22). However, the role of β2 integrins during acute or chronic schistosomiasis has not been investigated. Using a mice model that express low levels of CD18, we found that β_2_ integrin is important for lung accumulation of specific monocyte subsets, MDMs and MDCs after 7 days of infection with *S. mansoni*. Of importance, low CD18 expression results in monocytopenia in the peripheral blood and bone marrow early after infection, suggesting that proper CD18 expression is particularly required for monocyte hematopoiesis during an infectious process. After 48 days, CD18^low^ mice exhibited reductions in the percentage of neutrophils and absolute numbers of MDMs, as for increased levels of IFN-γ, TNF-α, and IL-10 in the lung. Intermediate and patrolling monocyte subsets were also reduced in the liver during chronic infection, while CD18 was required for proper parasite elimination and resistance against fatal outcomes. These data provide important insights into the immunopathogenesis of schistosomiasis and demonstrate a critical role of CD18 for the development and tissue accumulation of monocytes during infection.

## Materials and methods

### Mice

Male 12–15-week-old (22–26 g) C57BL/6 (WT) and homozygous *CD18*^*low*^ mice on the C57BL/6 background were obtained from the animal facilities of the Faculdade de Ciências Farmacêuticas de Ribeirão Preto – Universidade de São Paulo (FCFRP-USP), Brazil. The *CD18*^*low*^ (B6.129S-Itgb2^tm1bay^) mice were purchased at The Jackson Laboratory. All experiments using animals were approved by the Comissão de Ética no Uso de Animais da Faculdade de Ciências Farmacêuticas de Ribeirão Preto (Protocol Number 14.1.607.53.9) and carried out in accordance to the ethical principles for animal research adopted by the Sociedade Brasileira de Ciência em Animais de Laboratório.

### Parasite maintenance and experimental infection

*Schistosoma mansoni* LE strain was maintained by routine passage through *Biomphalaria glabrata* snails and BALB/c mice (20–25 g) from the animal facilities of the Faculdade de Medicina de Ribeirão Preto – Universidade de São Paulo (FMRP-USP). The infected snails were induced to shed cercariae under light exposure in water for 2 h. The number of cercariae in suspension was determined and mice were subcutaneously inoculated with 80 or 200 cercariae/animal with a sterile syringe and 22 G × 1″ needle (BD Biosciences, Franklin Lakes, New Jersey, USA). After 3, 7, 14, and 48 days post infection (dpi) the animals were euthanized for posterior analyses. For analysis of mice survival, mice were inoculated with 200 cercariae/animal with a sterile syringe and 22 G × 1″ needle (BD Biosciences, Franklin Lakes, New Jersey, USA) and monitored daily up to 70 dpi.

### Hepatic parasite burden, intestinal egg viability and fecal eggs quantification

Liver parasite burdens were assessed as previously described (23). Adult *S. mansoni* were collected from the hepatic portal system with PBS containing 0.02 U/ml heparin. The worms were washed and counted using a dissecting microscope. Intestinal egg viability was measured in fragments of the intestine (terminal ileum), as previously described (24). The fragments were examined with an optical microscope (100 ×), and 200 eggs/mouse were counted and classified according to the developmental stage as follows: *(i)* viable immature eggs (1st to 4th stage), *(ii)* viable mature eggs or *(iii)* dead eggs. The percentage of eggs in each egg stage was calculated. The Kato-Katz technique was used to quantify *S. mansoni* eggs in stool samples, as previously described (25).

### Flow cytometry of lung, liver, blood, and bone marrow cells

Lung cell suspensions were obtained after tissue digestion at 37°C for 45 min in 1 mL/lung digestion buffer [RPMI 1640, Liberase 0.05 mg/mL (Roche, Basel, Switzerland) and DNase 0.5 mg/mL (Sigma Aldrich, St. Louis, Missouri, USA)], as previously described (26). For analysis of liver cell populations, tissue fragment was collected and homogenized in 4 mL of digestion buffer [HBSS, 0.05% collagenase II (Sigma Aldrich, St. Louis, Missouri, USA) and 1 mg/mL DNase (Sigma Aldrich, St. Louis, Missouri, USA)] at 37°C for 45 min. The enzymatic digestion was stopped by adding 100 μL of FBS and the tissue fragments passed through a cell strainer 100 μm pore size (BD Biosciences, Franklin Lakes, New Jersey, USA). The resulting suspension was centrifuged at 1,300 rpm, 10 min, 4°C. The cellular pellet was resuspended in 40% of isotonic Percoll and centrifuged at room temperature for 30 min at 1,500 g. Next, red blood cells were lysed, and remaining cells were washed in PBS, centrifuged and resuspended in RPMI 1640 containing 5% FBS. Suspensions of 2 × 10^6^ cells from lung or liver were used in further analysis. Peripheral blood was drawn from the retro-orbital plexus. Bone marrow was flushed out from two femurs using RPMI. The red blood cells present in blood or bone morrow were lysed, and remaining cells were washed in PBS containing 5% FBS, centrifuged and resuspended in RPMI 1640 containing 5% FBS. Cell suspensions were used in further analysis. The following antibodies were used: CD11b (clone: M1/70); CD11c (clone: HL3); CD45 (clone: 30-F11); Ly6C (clone: HK1.4); Ly6G (clone: RB6-8C5); MHC-II (clone:M5/144.15.2), F4/80 (clone: BM8), CCR2 (clone: 5A203611) and CX3CR1 (clone: SA011F11). *In vivo* intravascular staining was performed as described (27). Briefly, 3μg of anti-CD45 antibody (Pacific Blue clone: 30-F11) were injected intravenously 3 minutes before euthanasia. The lung was processed for flow cytometry using a second anti-CD45 (APCCY7 clone: 30-F11), CD11b (clone: M1/70); CD11c (clone: HL3); Ly6C (clone: HK1.4); Ly6G (clone: RB6-8C5) and CX3CR1 (clone: SA011F11). All antibodies used for flow cytometry were purchased from eBioscience (San Diego, CA) or BD Biosciences (Franklin Lakes, New Jersey, USA). Data acquisition was performed using a FACSCanto II flow cytometer and FACSDiva software (BD Biosciences, Franklin Lakes, New Jersey, USA). 100,000 events were acquired for samples from lung, bone marrow and liver, while 50,000 events were acquired for blood samples. Data were plotted and analyzed using FlowJo software v.10.0.7 (Tree Star, Inc, Ashland, OR, USA).

### Cytokine quantification

Lungs from WT and CD18^low^ uninfected and *S. mansoni*-infected mice were removed, weighed, homogenized in H_2_O Milli-Q containing protease inhibitor (Complete, Roche, Basel, Switzerland) and centrifuged to remove cellular debris (1,500 rpm, 5 min, 4°C). Supernatants were collected and stored at −20°C. Levels of IFN-γ, IL-6, TNF-α, IL-4, IL-5, and IL-10 were measured by enzyme-linked immunosorbent assay (ELISA) according to the manufacturers' recommendations (R&D Systems, MN, USA and BD Pharmingen, San Jose, CA, USA).

### Lipid mediator quantification by LC-MS/MS

The lipid mediators LTB_4_ and PGE_2_ were measured in lungs from WT and CD18^low^ mice uninfected and infected with *S. mansoni*. The tissue was homogenized in methanol, centrifuged (800 g, 10 min, 4°C) and stored at −80°C. Supernatants were transferred to autosampler vials and 10 μL of each sample were injected on the liquid chromatography-tandem mass spectrometry (LC-MS/MS) system TripleTOF® 5600+ (AB Sciex - Foster, CA, USA), as previously described (28).

### Histopathological analysis

Animals from each experimental group were euthanized at 48 days post-infection (dpi). The liver was excised, fixed with 10% formalin for 24 h, and embedded in paraffin. The tissue sections (5μm) were stained with H&E coloration for histopathological evaluation. Images were captured with a digital video camera (Leica® Microsystems, Heebrugg, Switzerland) adapted to DMR microscope (Leica®, Microsystems GmbH, Wetzlar, Germany). The images were processed using the Leica QWin software (Leica Microsystems Image Solutions®, Cambridge, UK). The labeling area of granuloma was measured (around single eggs) in a horizontal plane using Image J software.

### Statistical analyses

The data are expressed as the medians ± interquartile range (IR). Significant differences between experimental groups along the course of the infection were evaluated with Kruskal-Wallis followed by Dunn's multi-comparison test and two tailed *p*-values are reported. Categorical comparisons between two experimental groups were performed with Mann-Whitney test and one-tailed *p*-values are reported. All analyses were performed with GraphPad Prism software v6.0 (GraphPad Software Inc., San Diego, CA, EUA). Statistical significance was set at *p* < 0.05.

## Results

### Low CD18 expression affects CD11b, but not CD11c expression by lung leukocytes during experimental schistosomiasis

**Figure 1:**
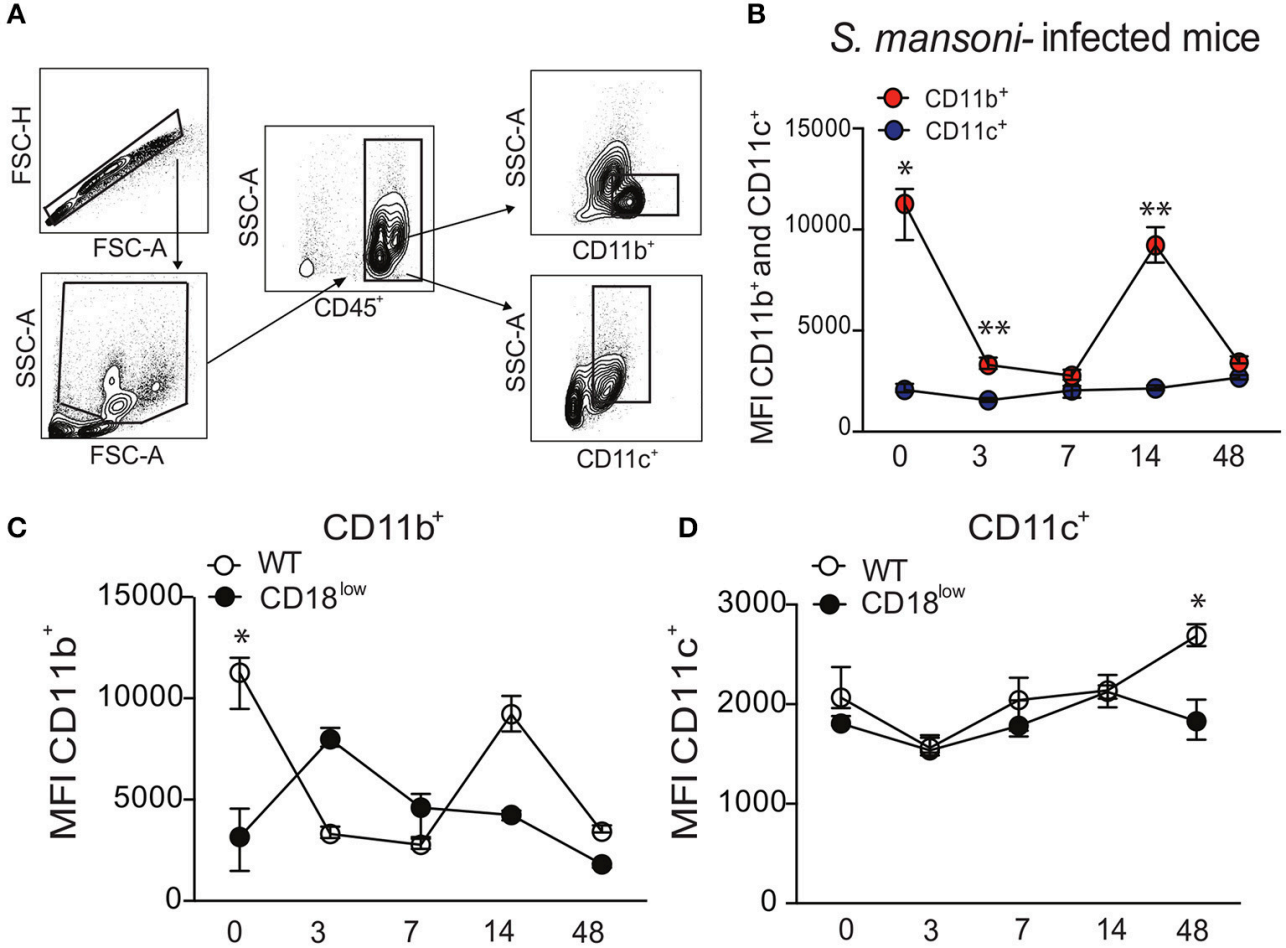
*S. mansoni* infection modulates the expression of integrin αM (CD11b) and αX (CD11c) subunits in lung leukocytes. Lungs of uninfected and *S. mansoni*-infected C57BL/6 and CD18^low^ mice were analyzed by flow cytometry. **(A)** Contour plots show representative flow cytometric data and indicate gating hierarchy for quantification of CD11b and CD11c expression. **(B)** Line plots show expression kinetics of CD11b or CD11c measured by mean fluorescence intensity (MFI) in lung leukocytes from C57BL/6 mice before and along the course of 48 days of infection. **(C,D)** Line plots show expression kinetics of CD11b **(C)** or CD11c **(D)** measured by MFI in lung leukocytes from C57BL/6 and CD18^low^ mice before and during 48 days of infection. Median with interquartile range are shown for representative data out of two independent experiments (*n* = 5–7 mice per group, at each time-point, in each experiment). ^*^p < 0.05, ^**^p < 0.01 compared between α subunits using Kruskal–Wallis followed by Dunn's multi-comparison test.

The common subunit of β_2_ integrins (CD18) partners with several α subunits, including CD11b or CD11c, to form functional adhesion molecules and receptors. To investigate whether β_2_ integrins play a role in lung response during *S. mansoni* infection, C57BL/6 (WT) mice were initially infected with 80 cercariae. Lung cells were isolated in different time points after infection, and leukocytes were evaluated for surface expression of the α subunits, CD11b or CD11c, using the flow cytometric gating hierarchy shown in Figure 1A. Lung leukocytes from naïve WT mice expressed higher levels of CD11b compared to CD11c, which were also significantly elevated 3 and 14 days after *S. mansoni* infection (Figure 1B). During 48 days of infection, CD11c expression remained unaltered in lung leukocytes, whereas CD11b expression was reduced at 3, 7, and 48 dpi compared to lung leukocytes from naïve mice (Figure 1B). Next, we evaluated whether low CD18 expression would alter the expression of α subunits in lung leukocytes during *S. mansoni* infection. WT or CD18^low^ mice were infected with 80 cercariae and the expression of CD11b and CD11c was evaluated by flow cytometry. Compared to naïve WT mice, CD11b expression was significantly reduced in leukocytes isolated from lungs of naïve CD18^low^ mice. After infection, cells from both experimental groups exhibited dynamic CD11b expression profiles throughout the 48 days of infection, but differences did not reach statistical significance (Figure 1C). CD11c expression was stable between the two groups until the 48th day of infection, when lung leukocytes from CD18^low^ mice exhibited significant reduction of CD11c expression (Figure 1D). Taken together, these results suggest that β_2_ integrins might play an important role for the host response during *S. mansoni* migration through the lung vasculature.

### CD18 promotes innate leukocyte accumulation in the lung during acute *S. mansoni* infection

**Figure 2:**
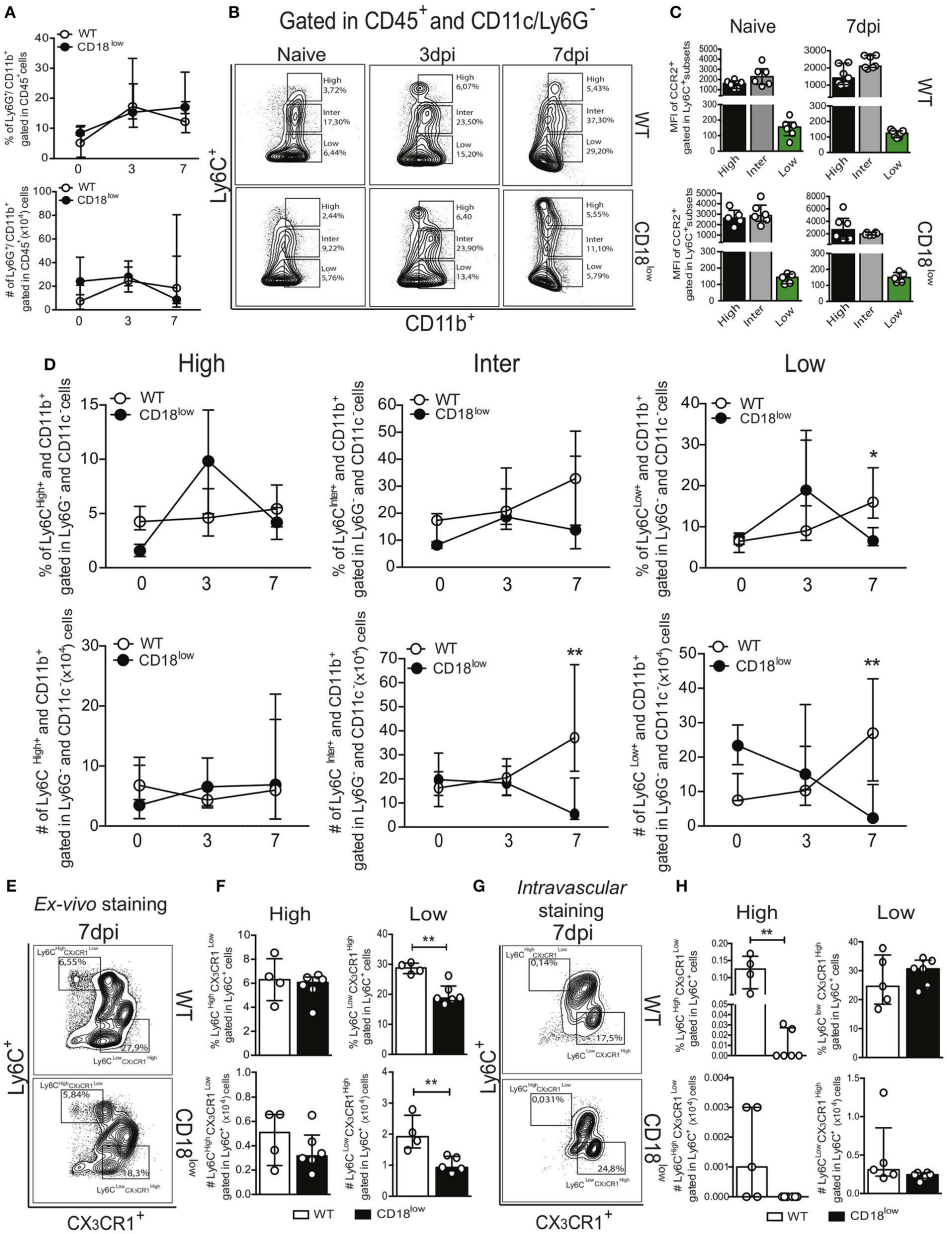
Low CD18 expression modulates specific monocyte subset accumulation in the lungs of *S. mansoni*-infected mice. Lungs of uninfected and *S. mansoni*-infected C57BL/6 and CD18^low^ mice were analyzed by flow cytometry. **(A)** Line plots showing the kinetics of percentage and absolute numbers of CD45^+^ CD11b^+^ Ly6G^+^ neutrophils before and along the course of 7 days of infection. **(B)** Contour plots show representative flow cytometric data of CD45^+^ CD11c^−^ Ly6G^−^ CD11b^+^ Ly6C^+^ monocyte subsets. **(C)** Scatter plot with bar show CCR2 mean fluorescence intensity (MFI) in cells expression varying levels of Ly6C before and 7 days after infection. **(D)** Line plots show kinetics of percentage and absolute numbers of distinct monocyte subsets. Median with interquartile range are shown for representative data of 4-6 uninfected-controls and 11–13 infected mice at 3 and 7 dpi. Results are a pool of two independent experiments. Data were analyzed with Kruskal–Wallis followed by Dunn's multi-comparison test (^*^p < 0.05, ^**^p < 0.01 compared to WT in each time-point). **(E)** Contour plots show representative flow cytometric data of distinct monocyte subsets, including the marker CX_3_CR1 **(F)** Scatter plot with bar show the percentage and absolute numbers of inflammatory Ly6C^high^ CX_3_CR1^low^ monocytes (upper gate) and patrolling Ly6C^low^ CX_3_CR1^high^ monocytes (lower gate). **(G)** Contour plots show representative flow cytometric data of distinct monocyte subsets, including the marker CX_3_CR1. **(H)** Scatter plot with bar show the percentage and absolute numbers of inflammatory Ly6C^high^ CX_3_CR1^low^ monocytes (upper gate) and patrolling Ly6C^low^ CX_3_CR1^high^ monocytes (lower gate) in the lung vasculature. Median with interquartile range are shown for data of 4-5 uninfected-controls and 6 infected mice at 7 dpi from one experiment. Data were analyzed with Mann-Whitney test (^*^p < 0.05, ^**^p < 0.01 compared to WT in each time-point).

During an infectious process, circulating myeloid cells are recruited for sites of inflammation and tissue damage by the action of chemokines, bioactive lipids, complement anaphylatoxins and adhesion molecules (13, 14). To determine the role of the common β_2_ subunit during *S. mansoni* migration through the lung, we evaluated the accumulation of innate immune leukocytes of infected WT or CD18^low^ mice early after infection. Along the course of 7 days of infection, there were no differences in the percentage or absolute number of neutrophils (Ly6G^+^) in the lungs (Figure 2A and Figure S1A). We also evaluated the accumulation of monocytes, which are subclassified by different levels of Ly6C expression: Ly6C^high^ (inflammatory monocytes), Ly6C^inter^ (intermediate monocytes) and Ly6C^low^ (patrolling monocytes) (Figure 2B and Figure S1B). These monocyte subsets display differential expression of the chemokine receptor CCR2 (29). Consistently, we observed that Ly6C^+^ subsets express high levels of CCR2, whereas patrolling LyC6^low^ monocytes express negligible levels of CCR2 (Figure 2C). We observed that both percentage and absolute number of inflammatory Ly6C^high^ monocytes remained unaltered in lungs of WT or CD18^low^ mice infected with *S. mansoni* (Figure 2D). However, the absolute number of intermediate Ly6C^inter^ monocytes was significantly reduced in the lungs of CD18^low^ animals at 7 dpi (Figure 2D). Moreover, both percentage and absolute number of patrolling Ly6C^low^ monocytes were significantly reduced in the lungs of CD18^low^ mice at 7 dpi (Figure 2D). These data suggest that CD18 regulates the accumulation of specific monocyte subsets in the lung early after *S. mansoni* infection. Inflammatory and patrolling monocytes also differ on the expression of the chemokine receptor CX_3_CR1, with patrolling monocytes expressing the highest levels (29). To gather further confidence that proper CD18 expression is required for patrolling monocyte accumulation in the lung early after *S. mansoni* infection, we evaluated these cells in the lungs of WT and CD18^low^ mice infected with *S. mansoni* for 7 days but including the monocyte phenotypic marker CX_3_CR1 (Figure 2E and Figure S1C). Corroborating our previous analysis, inflammatory Ly6${\text{C}}^{\text{high}}CX_{3}CR1^{\text{low}}$ monocytes remained unaltered in the lungs of WT and CD18^low^ mice (Figure 2F). In contrast, both percentage and absolute numbers of patrolling Ly6${\text{C}}^{\text{low}}{\text{CX}}_{3}CR1^{\text{high}}$ monocytes were reduced in the lungs of CD18^low^ mice at 7dpi (Figure 2F).

Patrolling Ly6C^low^ CX_3_CR1^high^ monocytes actively survey the vascular endothelium in a CD18-dependent manner and rapid invade tissues upon sterile inflammation and infection (12). Although schistosomula circulate through the lung, they do not actively transmigrate to the parenchyma, but rather accumulate in capillaries where they cause tissue damage due their large size (30). Therefore, it is possible that patrolling monocytes were reduced in the lung capillaries instead of the lung parenchyma. To test this hypothesis, we performed intravascular staining using anti-CD45 to track leukocytes present in the lung capillaries of WT and CD18^low^ mice infected with *S. mansoni* for 7 days (Figure 2G and Figure S1C). Interestingly, the percentage of inflammatory Ly6C^high^ CX_3_CR1^low^ monocytes from CD18^low^ mice was reduced in the lung vasculature when compared to WT mice (Figure 2H). However, these cells were greatly underrepresented in lung vasculature of both mouse strains when compared to those that infiltrated the lung parenchyma (Figure 2F). This indicates that inflammatory monocytes have infiltrated the lung tissue. In contrast, patrolling Ly6C^low^ CX_3_CR1^high^ monocytes were equally represented in the lung vasculature of WT and CD18^low^ mice (Figure 2H), demonstrating that low CD18 expression affects the infiltration of specific monocyte subsets in the lung tissue early after infection with *S. mansoni*.

**Figure 3:**
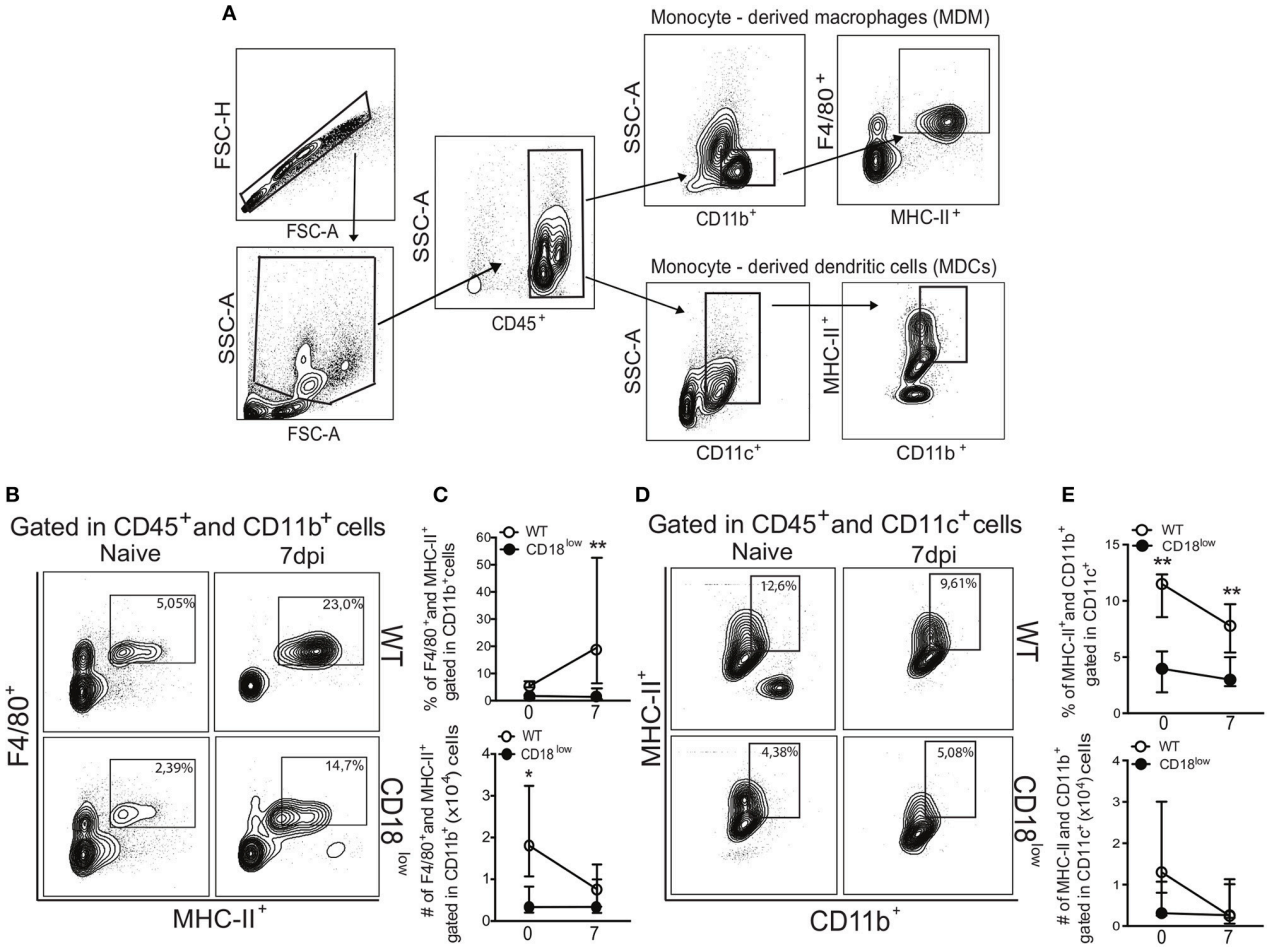
Low CD18 expression reduces the frequency of monocyte-derived macrophages and dendritic cells in the lungs of *S. mansoni*-infected mice. **(A)** Contour plots show representative flow cytometric gating hierarchy for analysis of CD45^+^ CD11b^+^ F4/80^+^ MHC-II^+^ monocyte derived macrophage (MDMs) and CD45^+^ CD11c^+^ MHC-II^+^ CD11b^+^ monocyte derived dendritic cells (MDCs). **(B)** Contour plots show representative flow cytometric data of MDMs. **(C)** Line plots show kinetics of percentage and absolute numbers of MDMs. **(D)** Contour plots show representative flow cytometric data of MDCs. **(E)** Line plots show kinetics of percentage and absolute numbers of MDCs. Median with interquartile range are shown for representative data of 6 uninfected-controls and 11–13 infected mice at 7 dpi and result are a pool of two independent experiments. Data were analyzed with Kruskal–Wallis followed by Dunn's multi-comparison test (^*^p < 0.05, ^**^p < 0.01, compared to WT in each time-point).

Once they infiltrate into inflammatory foci, monocytes can differentiate into MDMs or MDCs (10), which are characterized mainly by the expression of the surface markers F4/80 and CD11c, respectively (Figure 3A). Compared to naïve WT mice, absolute numbers of pulmonary MDMs were reduced in naïve CD18^low^ animals, but similar at 7 dpi (Figure 3C). Despite of that, the percentage of pulmonary MDMs was significantly higher in WT mice compared to CD18^low^ animals (Figures 3B,C), whose percentage and absolute numbers of MDMs were already low before infection and remained unchanged at 7 dpi (Figure 3C). Furthermore, we observed that the percentage of pulmonary MDCs were significantly reduced in CD18^low^ mice, both before and after 7 days of infection with *S. mansoni* (Figures 3D,E). Taken together, these data suggest that impaired infiltration of specific monocyte subsets in the lungs of CD18^low^ mice also impacts the accumulation of MDMs and MDCs early after infection with *S. mansoni*.

### CD18 regulates monocyte hematopoiesis during acute *S. mansoni* infection

**Figure 4:**
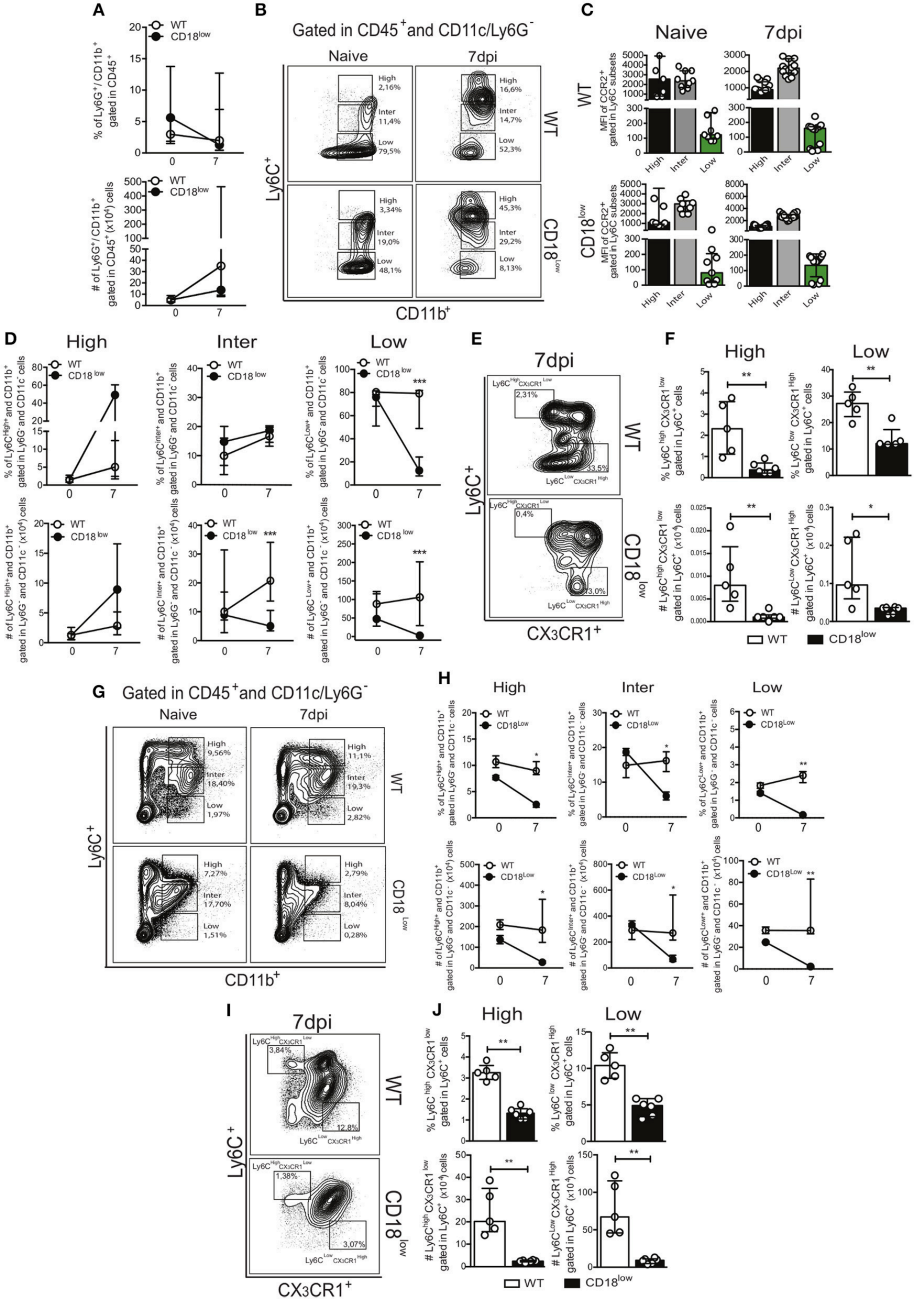
Low CD18 expression impacts monocytopoiesis during acute *S. mansoni* infection. Peripheral blood and bone marrow of uninfected and *S. mansoni*-infected C57BL/6 and CD18^low^ mice were analyzed by flow cytometry. **(A)** Line plots show the percentage and absolute numbers of neutrophils before and after 7 days of infection. **(B)** Contour plots show representative flow cytometric data of CD45^+^ CD11c^−^ Ly6G^−^ CD11b^+^ Ly6C^+^ monocyte subsets in the peripheral blood. **(C)** Scatter plot with bar show CCR2 mean fluorescence intensity (MFI) in cells expression varying levels of Ly6C before and 7 days after infection. **(D)** Line plots show the percentage and absolute numbers of distinct monocyte subsets in the peripheral blood. **(E)** Contour plots show representative flow cytometric data of distinct monocyte subsets in the peripheral blood, including the marker CX_3_CR1. **(F)** Scatter plot with bar show the percentage and absolute numbers of inflammatory Ly6C^high^ CX_3_CR1^low^ monocytes (upper gate) and patrolling Ly6C^low^ CX_3_CR1^high^ monocytes (lower gate) in the peripheral blood. Median with interquartile range are shown for representative data of 9 uninfected-controls and 15–17 infected mice at 7 dpi and results are a pool of two independent experiments. Data were analyzed with Kruskal-Wallis followed by Dunn's multi-comparison test or Mann-Whitney test (^*^p < 0.05, ^**^p < 0.01, ^***^p < 0.001 compared to WT in each time-point). **(G)** Contour plots show representative flow cytometric data of CD45^+^ CD11c^−^ Ly6G^−^ CD11b^+^ Ly6C^+^ monocyte subsets in the bone marrow. **(H)** Line plots show the percentage and absolute numbers of distinct monocyte subsets in the bone marrow**. (I)** Contour plots show representative flow cytometric data of distinct monocyte subsets in the bone marrow, including the marker CX_3_CR1. **(J)** Scatter plot with bar show the percentage and absolute numbers of inflammatory Ly6C^high^ CX_3_CR1^low^ monocytes (upper gate) and patrolling Ly6C^low^ CX_3_CR1^high^ monocytes (lower gate) in the bone marrow. Median with interquartile range are shown for representative data of one independent experiment (*n* = 2 uninfected-controls and 5-6 infected mice per group at 7 dpi). Data were analyzed with Kruskal–Wallis followed by Dunn's multi-comparison test or Mann-Whitney (^*^p < 0.05, ^**^p < 0.01, compared to WT in each time-point).

Lower accumulation of specific monocyte subsets in lungs of CD18^low^ mice suggest that they were unable to properly infiltrate the tissue, and thus would remain in the vasculature. Although the frequency of patrolling Ly6C^low^ monocytes was similar in lung vasculature of WT and CD18^low^ mice infected with *S. mansoni* for 7 days, we hypothesized that these cells would thus increase in the peripheral circulation. Therefore, we analyzed the frequency of neutrophils and monocytes in the whole blood of WT and CD18^low^ mice early after infection. Percentage and absolute number of neutrophils were similar between both mouse strains (Figure 4A). To investigate blood monocytes, we first applied the flow cytometric gating hierarchy shown in Figure 4B and Figure S1B, which also revealed monocyte subset-dependent CCR2 expression (Figure 4C). There were no significant differences in inflammatory Ly6C^high^ monocytes between both mouse strains (Figure 4D). Surprisingly, we observed that absolute numbers of intermediate Ly6C^inter^ monocytes and both percentage and absolute numbers of patrolling Ly6C^low^ monocytes were also reduced in the blood of infected CD18^low^ mice (Figure 4D). We thus proceeded with the analysis using a flow cytometric gating hierarchy to include the CX_3_CR1 marker (Figure 4E and Figure S1D). Interestingly, we confirmed that patrolling Ly6C^low^ CX_3_CR1^high^ monocytes were indeed reduced in the peripheral blood at 7 dpi (Figure 4F). However, this analysis revealed that inflammatory Ly6^high^ CX_3_CR1^low^ were also reduced in the peripheral blood (Figure 4F). Since β_2_ integrins are major regulators of trans-endothelial migration, we sought to investigate whether CD18 was necessary for monocyte egress from the bone marrow. For that, we evaluated monocytes in the bone marrow of WT and CD18^low^ mice after 7 days of infection with *S. mansoni*. Strikingly, both percentage and absolute numbers of all monocyte subsets were reduced in the bone marrow of CD18^low^ mice at 7dpi (Figures 4G,H), a phenomenon that was also observed when monocytes were characterized by CX_3_CR1 expression (Figures 4I,J). Taken together, reductions of monocytes in the peripheral blood and bone marrow suggest that low CD18 expression impairs the monocytic hematopoietic compartment during *S. mansoni* infection.

### Low CD18 expression impacts innate leukocyte accumulation in the lung and liver during chronic schistosomiasis

**Figure 5:**
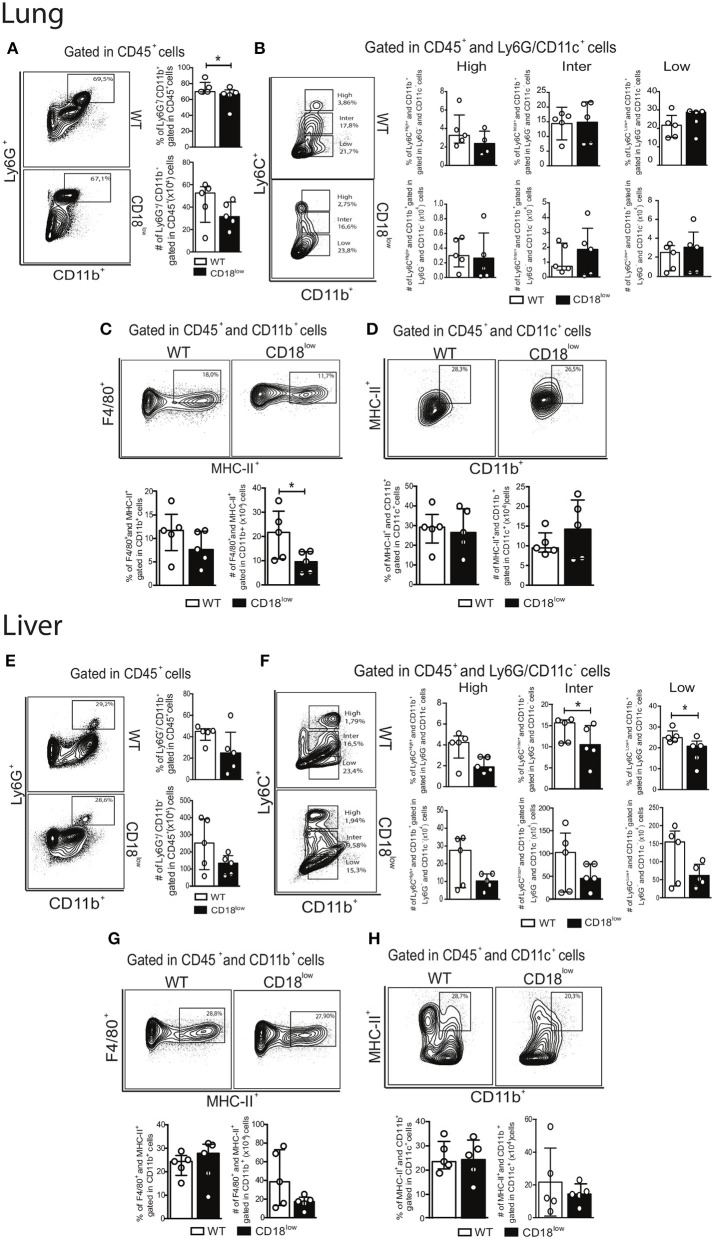
CD18 is required for accumulation of specific leukocyte subsets in the lungs and liver during chronic schistosomiasis. Lungs and Livers of *S. mansoni*-infected C57BL/6 and CD18^low^ mice were analyzed by flow cytometry at 48 dpi. **(A)** Contour plots show representative flow cytometric data of CD45^+^ CD11b^+^ Ly6G^+^ neutrophils and scatter plots with bar show the percentage and absolute numbers of neutrophils in the lung. **(B)** Contour plots show representative flow cytometric data of CD45^+^ CD11c^−^ Ly6G^−^ CD11b^+^ Ly6C^+^ monocyte subsets and scatter plots with bar show the percentage and absolute numbers of distinct monocyte subsets in the lung. **(C)** Contour plots show representative flow cytometric data of MDMs and scatter plot with bar show the percentage and absolute numbers of these cells in the lung **(D)** Contour plots show representative flow cytometric data of MDCs and scatter plot with bar show of percentage and absolute numbers of these cells in the lung. Median with interquartile range are shown for representative data of 4-5 WT and CD18^low^ infected mice at 48 dpi and results are from one independent experiment. Data were analyzed with Mann-Whitney test (^*^p < 0.05, ^**^p < 0.01, compared to WT in each time-point). **(E)** Contour plots show representative flow cytometric data of CD45^+^ CD11b^+^ Ly6G^+^ neutrophils and scatter plot with bar show the percentage and absolute numbers of neutrophils in the liver. **(F)** Contour plots show representative flow cytometric data of CD45^+^ CD11c^−^ Ly6G^−^ CD11b^+^ Ly6C^+^ monocyte subsets and scatter plot with bar show the absolute numbers of distinct monocyte subsets in the liver. **(G)** Contour plots show representative flow cytometric data of MDMs and scatter plot with bar show the percentage and absolute numbers of these cells in the liver. **(H)** Contour plots show representative flow cytometric data of MDCs and scatter plot with bar show the percentage and absolute numbers of these cells in the liver. Data are from one experiment (*n* = 5 WT and CD18^low^ infected mice at 48 dpi) and were analyzed with Mann-Whitney test (^*^p < 0.05 compared to WT in each time-point).

During chronic stages of the disease, mature parasites release eggs that trigger the granulomatous response in affected tissues (31). We thus evaluated the accumulation of innate leukocytes in the lungs of WT and CD18^low^ mice infected with *S. mansoni* for 48 days. We observed a slight reduction in the percentage of neutrophils (Figure 5A), as for a reduction of the absolute number of MDMs in the lungs of CD18^low^ mice (Figure 5C). However, the remaining cellular populations were unaltered between mice strains (Figures 5B,D). Formation of granulomas around eggs requires leukocyte recruitment into the liver, including inflammatory Ly6C^high^ and patrolling Ly6C^low^ monocytes (16). We thus sought to investigate whether CD18 is necessary for efficient accumulation of leukocytes in the liver after 48 days of infection with *S. mansoni*. We found that lower CD18 expression does not affect neither neutrophil nor MDM or MDC frequency or absolute numbers in livers of *S. mansoni*-infected mice (Figures 5E,G,H). However, the percentages of intermediate Ly6C^inter^ and patrolling Ly6C^low^ monocytes were reduced in the livers of CD18^low^ mice, while inflammatory Ly6C^high^ monocytes were not significantly altered compared to WT animals (Figure 5F). These results suggest that impaired monocyte hematopoiesis in CD18^low^ mice affects the accumulation of specific monocyte subsets during chronic infection with *S. mansoni*.

### CD18 regulates cytokine production in the lung during *S. mansoni* infection

**Figure 6:**
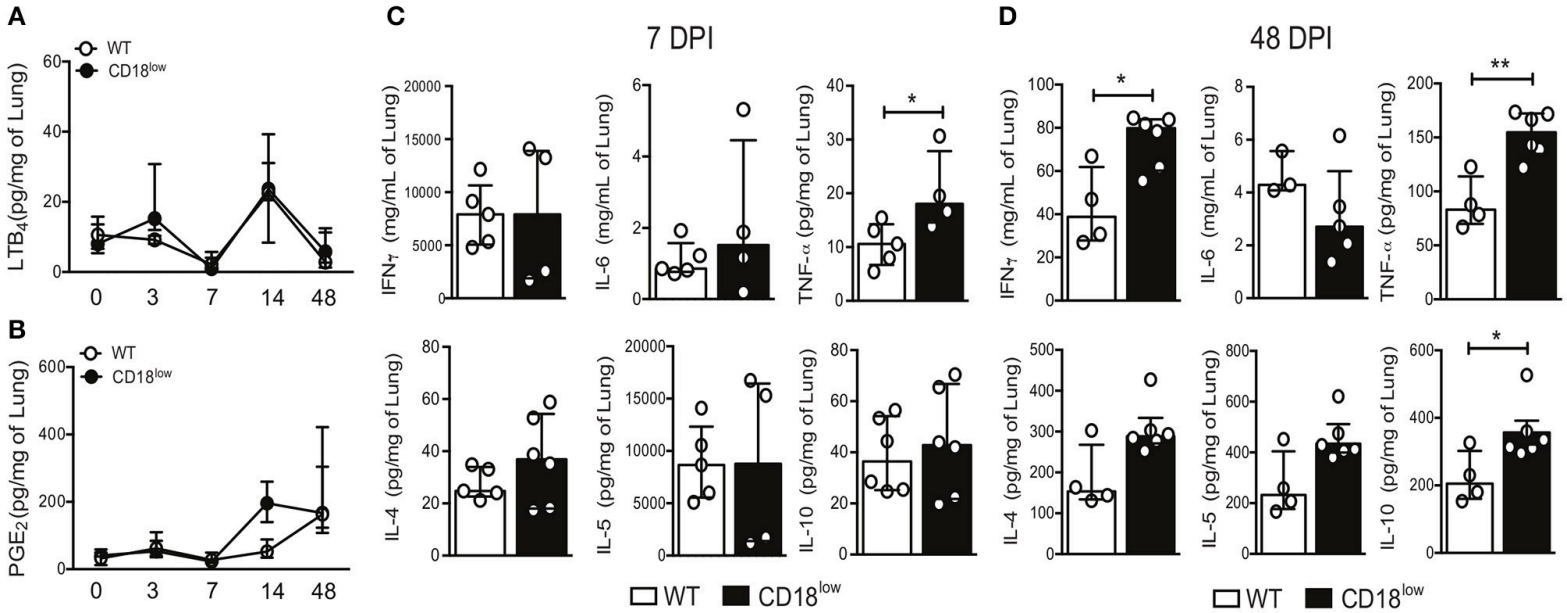
CD18 is required for regulated production of cytokines in lungs of *S. mansoni*-infected mice. Lungs of uninfected and *S. mansoni*-infected C57BL/6 and CD18^low^ mice were analyzed by liquid-chromatography tandem mass spectrometry (LC-MS/MS) and immunoenzymatic assay (ELISA). **(A)** Line plots show kinetics of LTB_4_ quantification by LC-MS/MS. **(B)** Line plots show kinetics of PGE_2_ quantification by LC-MS/MS. Median with interquartile range are shown for one independent experiment (*n* = 3–5 mice per group at each time-point). Data were analyzed with Kruskal-Wallis followed by Dunn's multi-comparison test. **(C,D)** Scatter plots with bar show quantification of IFN-γ, IL-6, TNF-α, IL-4, IL-5, and IL-10 by ELISA at 7 dpi **(C)** and 48 dpi **(D)**. Median with interquartile range are shown for one independent experiment (*n* = 4–6 WT and CD18^low^ infected mice at 7 and 48 dpi) and were analyzed with Mann-Whitney test (^*^p < 0.05, ^**^p < 0.01 compared to WT in each time-point).

The production of eicosanoids by monocytes, such as LTB_4_, induces β_2_ integrin-dependent adhesion (13), while 5-lipoxygenase, a rate limiting enzyme for the production of leukotrienes, is crucial for the efficient formation of lung granulomas induced by *S. mansoni* eggs (31). We thus quantified LTB_4_ and PGE_2_ in lungs WT and CD18^low^ mice over the course of 48 days of *S. mansoni* infection. Of note, there were no significant differences on PGE_2_ or LTB_4_ levels between the experimental groups (Figures 6A,B).

During immature stages of *S. mansoni* on the mammalian host, immune cells recognize parasite antigens and initiate the production of cytokines such as IFN-γ, IL-6, TNF-α, but once parasites mature and lay eggs, this profile changes toward production of IL-4, IL-5, and IL-10 (7). To elucidate the impact of CD18 for lung cytokine profiles during early and later phases of the infection, WT and CD18^low^ mice were infected with 80 cercariae and lungs were collected after 7 and 48 dpi. At an early stage of infection (7 dpi), only TNF-α levels were significantly increased in lungs of CD18^low^ compared to WT mice (Figure 6C). Interestingly, even after the parasite passage through the lung and maturation in liver and gut, CD18^low^ mice showed increased levels of IFN-γ, TNF-α, and IL-10 at 48 dpi (Figure 6D). These data indicate that CD18 impacts significantly the function of immune cells in the lungs during *S. mansoni* infection. They affect not only cellular accumulation but are also required for the balance in cytokine production during acute and chronic schistosomiasis.

### CD18 confers resistance against experimental *S. mansoni* infection

**Figure 7:**
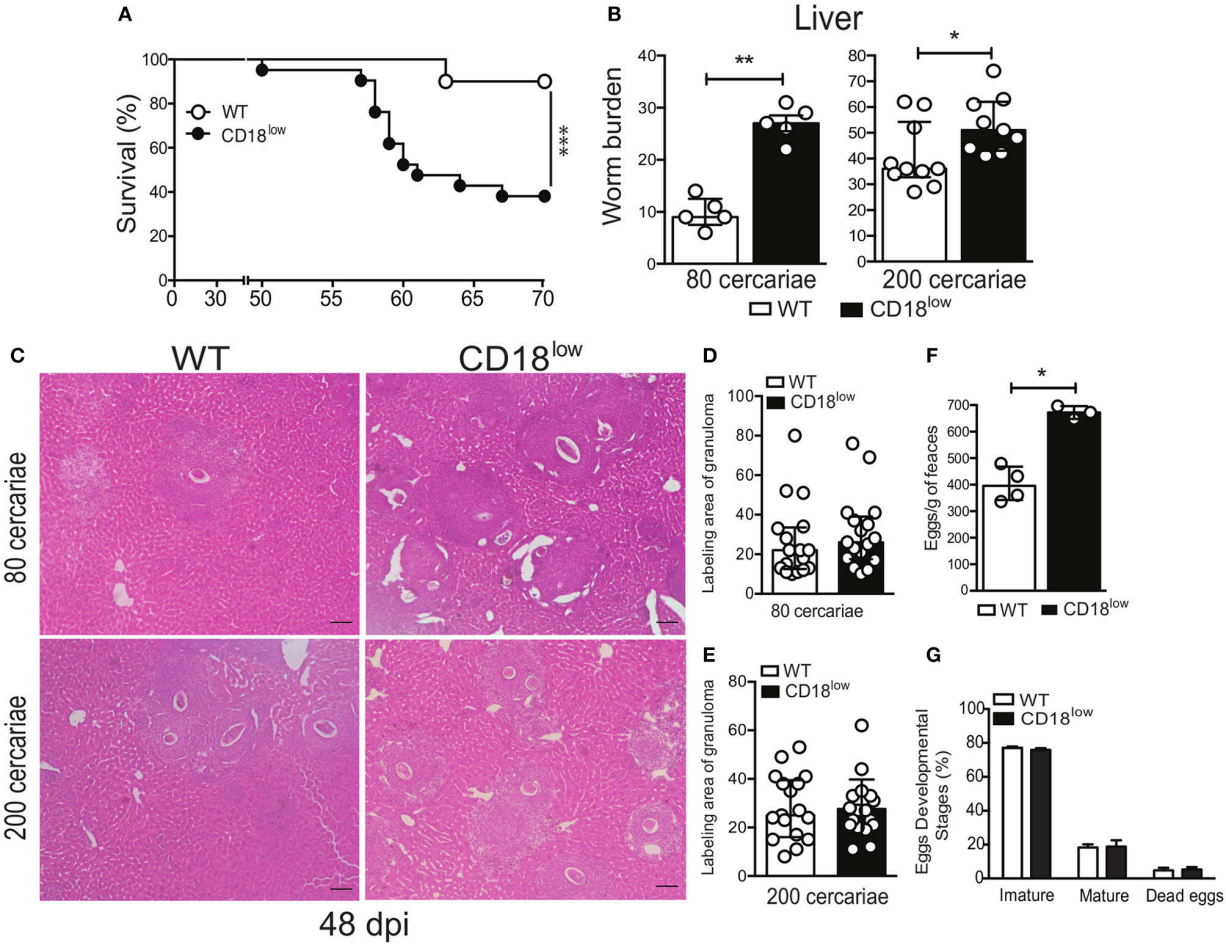
Low CD18 expression promotes susceptibility to *S. mansoni* infection. **(A)** Line plots show survival of WT and CD18^low^ mice were infected subcutaneously with 200 cercariae of *S. mansoni* and monitored daily for 70 days (*n* = 13 per group). ^***^p < 0.001 using log-rank test. **(B)** Scatter plot with bar show the parasite worm burden determined by perfusion of the hepatic portal system on the 48th day after infection with 80 (*n* = 5 per group) and 200 cercariae (*n* = 10 WT and 9 CD18^low^ infected mice). **(C)** Photomicrographs of liver lesion by H&E coloration (original magnification, X100) from WT and CD18^low^ mice infected with 80 and 200 cercariae at 48 dpi with *S. mansoni*. **(D,E)** Scatter plots with bar show the labeling area of liver granuloma at 48 dpi with 80 cercariae and 200 cercariae, respectively. **(F)** Bar plot show mean ± SEM of eggs/g of feces from WT and CD18^low^ mice infected with 80 cercariae of *S. mansoni* at 48 dpi, quantified according to Kato-Katz technique (*n* = 4 WT and 3 CD18^low^ infected mice). **(G)** Bar plots show the percentage of immature, mature or dead *S. mansoni* eggs in the intestinal tissue from C57BL/6 and CD18^low^ mice infected with 80 cercariae of *S. mansoni* at 48 dpi (*n* = 3 per group). The intestinal eggs were identified using the oogram methodology. Median with interquartile range are shown for one representative experiment out of two independent experiments. Data were analyzed with Mann-Whitney test (^*^p < 0.05, ^**^p < 0.01 compared to WT in each time-point).

To assess the importance of CD18 during chronic stages of the infection, WT and CD18^low^ mice were infected with 200 cercariae and survival was monitored for up to 70 dpi. Of note, lower CD18 expression resulted in enhanced fatal outcomes to *S. mansoni* infection, as 61.9% of the animals succumbed within 70 dpi, compared to 10% of WT mice (Figure 7A). To confirm that this effect was independent of the initial parasite inoculum, CD18^low^ and WT mice were infected with two different parasite inoculums (80 or 200 cercariae) and 48 dpi the animals was euthanized to quantify the parasite burden in the hepatic portal system. Independently of the initial parasite inoculum, CD18^low^ mice had increased worm burdens in the livers at 48 dpi when compared to WT animals (Figure 7B).

During chronic infections with *S. mansoni*, granulomas develop in the lung and liver to contain eggs that reach the circulation and tissues (7, 32). To assess whether CD18 is important for the granulomatous response, livers from CD18^low^ and WT mice were collected at 48 dpi, after infection with 80 or 200 cercariae. Tissue staining with hematoxylin & eosin (H&E) showed that CD18^low^ mice presented greater number of granulomas around eggs that spread all over the tissue (Figure 7C). However, granuloma areas were similar between the experimental groups (Figures 7D,E). This result suggests higher egg deposition by mature *S. mansoni* in CD18^low^ mice compared to WT animals. To validate these findings, we assessed eggs on feces of animals from both groups. Accordingly, CD18^low^ mice displayed increased number of eggs in feces at 48 dpi (Figure 7F). However, there were no differences on egg maturation and viability (Figure 7G). Overall, these data demonstrate that CD18 is required for specific leukocyte accumulation, proper granuloma formation, and parasite clearance during chronic schistosomiasis. Overall, these data suggest that increased tissue pathology caused by unbalanced cellular and cytokine profile in the lung, as well greater numbers of liver granulomas and consequent tissue damage, culminates in higher susceptibility of CD18^low^ mice to experimental schistosomiasis.

## Discussion

Schistosomiasis is a neglected parasitic disease caused by *Schistosoma* spp. worms, which affects mainly children of tropical and subtropical regions (33). Severe symptoms include liver damage, pulmonary hypertension and even pericarditis (4, 5, 34). During infection of mammalian hosts, schistosomula migrate through the pulmonary-systemic vasculature before they reach the hepatic portal system (3). While migrating through the lung, some schistosomula are blocked by infiltrating leukocytes or even disrupt blood vessels and enter the alveoli, from which they are unable of return to circulation (30). This results in a subtle inflammatory reaction, mostly considered as a tissue damage repair response. However, the dynamics of specific leukocyte accumulation in the lung during schistosomula migration is unknown. In this study, we identified a critical role of the common subunit of β_2_ integrins for efficient accumulation of intermediate and patrolling monocytes in the lung early after infection. Of note, patrolling Ly6C^low^ monocytes express high levels of lymphocyte function-associated antigen 1 (LFA-1 – CD11a/CD18) integrin and depend on this adhesion molecule to crawl on the endothelial wall in a steady state. Our study expands this knowledge by demonstrating that CD18 is also required for specific monocyte subset infiltration into the lung during an inflammatory process. Reduction of these monocyte subsets was associated with diminished percentage of MDMs and MDCs and increased levels of TNF-α, which remained elevated in the lung 48 days after infection. These data suggest that reduction of specific innate leukocytes in the lung early after infection might result in a deregulated inflammatory response that persists over time, even though the parasites are not there. This is plausible because acute infections can disrupt the communication between tissues and the immune system, impairing immune cell functions (35).

Strikingly, we found that low CD18 expression causes monocytopenia in the bone marrow and peripheral blood after 7 days of infection, which would explain the reduction of specific monocyte subsets in the lung. However, intravascular leukocyte staining demonstrated that while inflammatory Ly6C^high^ monocytes do not depend on CD18 to exit lung capillaries and enter the lung tissue, patrolling Ly6C^low^ monocytes were unable to do so, suggesting that β_2_ integrin also controls trans-endothelial migration of these cells. Nevertheless, intermediate and patrolling monocytes were also reduced in the liver during chronic infection of CD18^low^ mice. Their livers contained greater numbers of granulomatous lesions and increased parasite burden, suggesting that CD18^low^ mice exhibit a defective monocytic hematopoietic compartment and possible dysfunction of protective effector and regulatory mechanisms. In line with this hypothesis, human intermediate CD14^bright^ CD16^+^ monocytes present with an enhanced ability to bind to cercarial and egg excretory/secretory products, implicating these cells in *Schistosoma* recognition by the innate immune system (36). Inflammatory Ly6C^high^ monocytes are recruited to the liver by the axis CCR2/CCL2 and favor a protective environment (15, 16). Indeed, these cells differentiate into alternatively activated macrophages (AAM) (15, 16), which protect from hepatocellular damage and mediate survival during experimental schistosomiasis (37). Of interest, differentiation of inflammatory Ly6C^high^ monocytes into AAM seems to transition through a Ly6C^low^ state during chronic *S. mansoni* infection (16). Although we have not observed differences in the frequency of MDMs in the liver, these data suggest that CD18 could also be required for the differentiation of inflammatory Ly6C^high^ monocytes into AAM and regulate the granulomatous response around eggs. Interestingly, a recent study demonstrated that patrolling Ly6C^low^ monocytes that developed from monocytic precursors in the bone marrow, give rise to AAM in the lung and protect from influenza-induced pathology (38). This highlights the potential of patrolling Ly6C^low^ monocytes to differentiate into AAM and protect from tissue damage caused by schistosomula migration through the lung. Future studies will be necessary to determine the molecular cues controlled by CD18 during monocytopoesis and further differentiation. Of importance, low CD18 expression has been shown to induce an expansion of hematopoietic stem cells (39), which could impact the development of monocytes during an inflammatory process.

Polymorphonuclear leukocytes, such as neutrophils, also express the β_2_ integrins CD11b/CD18 (Mac1 or CR3) and CD11a/CD18 (LFA-1) (40). Mac1/CR3 was associated with neutrophil and eosinophil recruitment after stimulus with extracts of *S. mansoni* larvae in guinea pig model (21). However, in the mouse model of *S. mansoni* infection, we show that neutrophils (Ly6G^+^) infiltrate the lung even in conditions of low CD18 expression. This indicates that neutrophils are activated and migrate to the affected tissues independently of β_2_ integrins. Beyond cell adhesion and trans-endothelial migration, β_2_ integrins display intracellular signaling capacities, which seem to be important during experimental schistosomiasis. This hypothesis arises from the observation that low CD18 expression has a significant impact on the production of TNF-α in the lung early after infection. TNF-α is important to induce expression of adhesion molecules by endothelial cells (41), thus increased TNF-α levels could reflect a compensatory mechanism due low CD18 expression. We observed increased levels of INF-γ, TNF-α, and IL-10 long after parasites passed through the lungs of CD18^low^ mice, possibly due a deregulated T lymphocyte response. These results suggest that low CD18 expression may also affect T lymphocyte function and promote a systemic inflammatory imbalance due failures in parasite elimination.

The granulomatous response is crucial to protect against a diversity of pathogens such as the fungus *Paracoccidioides brasiliensis* (42), the intracellular parasite *Leishmania donovani* (23), and *S. mansoni* (31). We observed that low CD18 expression did not impair the formation of granulomas around eggs during chronic infection. However, CD18^low^ mice displayed greater numbers of granulomatous lesions that were unable to eliminate parasites efficiently, reflected by increased worm burden and egg counts in the feces. Consistent with these data, we also observed higher mortality of CD18^low^ mice at the end of 70 days of infection. Therefore, the common subunit of β_2_ integrins is crucial for resistance to *S. mansoni* infection. This could be determined during early schistosomula migration through the lung vasculature, where efficient parasite elimination would lower liver burden at later stages. Supporting this hypothesis, previous studies indicate that the lungs are the major site of worm elimination, both in normal and mice vaccinated with irradiated cercariae (30, 43). However, one limitation of our study is given by the route of parasite inoculation. Penetration of cercariae in the skin results in significant alterations in the larvae physiology and biochemistry. Skin-stage schistosomula are susceptible to the host immune response, but rapid develop resistance to humoral and cellular immunity (44), indicating that parasites inoculated by percutaneous or subcutaneous routes may induce distinct host responses. This is particularly relevant for our study, as autoradiographic analysis demonstrated that fewer parasites inoculated by percutaneuous route reach the lungs and decline faster when compared to the subcutaneous route (45). However, this does not seem to cause a significant difference on the recovery of parasites in the liver after chronic infection (46). Moreover, we believe our findings to be highly relevant to individuals with leukocyte adhesion deficiency type-I, a primary immunodeficiency caused by mutations on the *ITGB2* gene which encodes the common β_2_ integrin subunit in humans (47). These individuals present recurrent infections (48), whereby data presented here also implicates in higher susceptibility to helminth infections.

In summary, this study demonstrates the critical role of β_2_ integrins during experimental *S. mansoni* infection, providing important insights into host responses promoted by these molecules during schistosomiasis. Further investigation is necessary to uncover the specific α subunits, and thus functional integrins, that are responsible for the phenomena describe herein. Importantly, our study raises novel perspectives about the role of specific monocyte subsets during acute and chronic schistosomiasis.

## Author contributions

CS, ME, FF, and LF conceived the study. CS, ME, CF, MP, and LG performed experiments. CS, ME and LG conduced data analysis. VR maintained parasites and provided infection model. CS, LG, and LF wrote the paper. LF and LG supervised the study. All authors read and approved the final manuscript.

### Conflict of interest statement

The authors declare that the research was conducted in the absence of any commercial or financial relationships that could be construed as a potential conflict of interest.
