# Supplementary material for: CD18 Regulates Monocyte Hematopoiesis and Promotes Resistance to Experimental Schistosomiasis
Source: Front Immunol. 2018 Aug 31;9:1970. doi: 10.3389/fimmu.2018.01970 (PMC6127275; doi:10.3389/fimmu.2018.01970)
Supplement: Supplementary file 1 [file Image_1.pdf]

Supplementary Material

CD18 regulates monocyte hematopoiesis and promotes resistance to experimental schistosomiasis

Camila Oliveira Silva Souza1¶, Milena Sobral Espíndola1¶,#a, Caroline Fontanari1, Morgana Kelly B. Prado1, Fabiani Gai Frantz1, Vanderlei Rodrigues2, Luiz Gustavo Gardinassi1, Lúcia Helena Faccioli1*

*** Correspondence:**
Lúcia Helena Faccioli, PhD
faccioli@fcfrp.usp.br

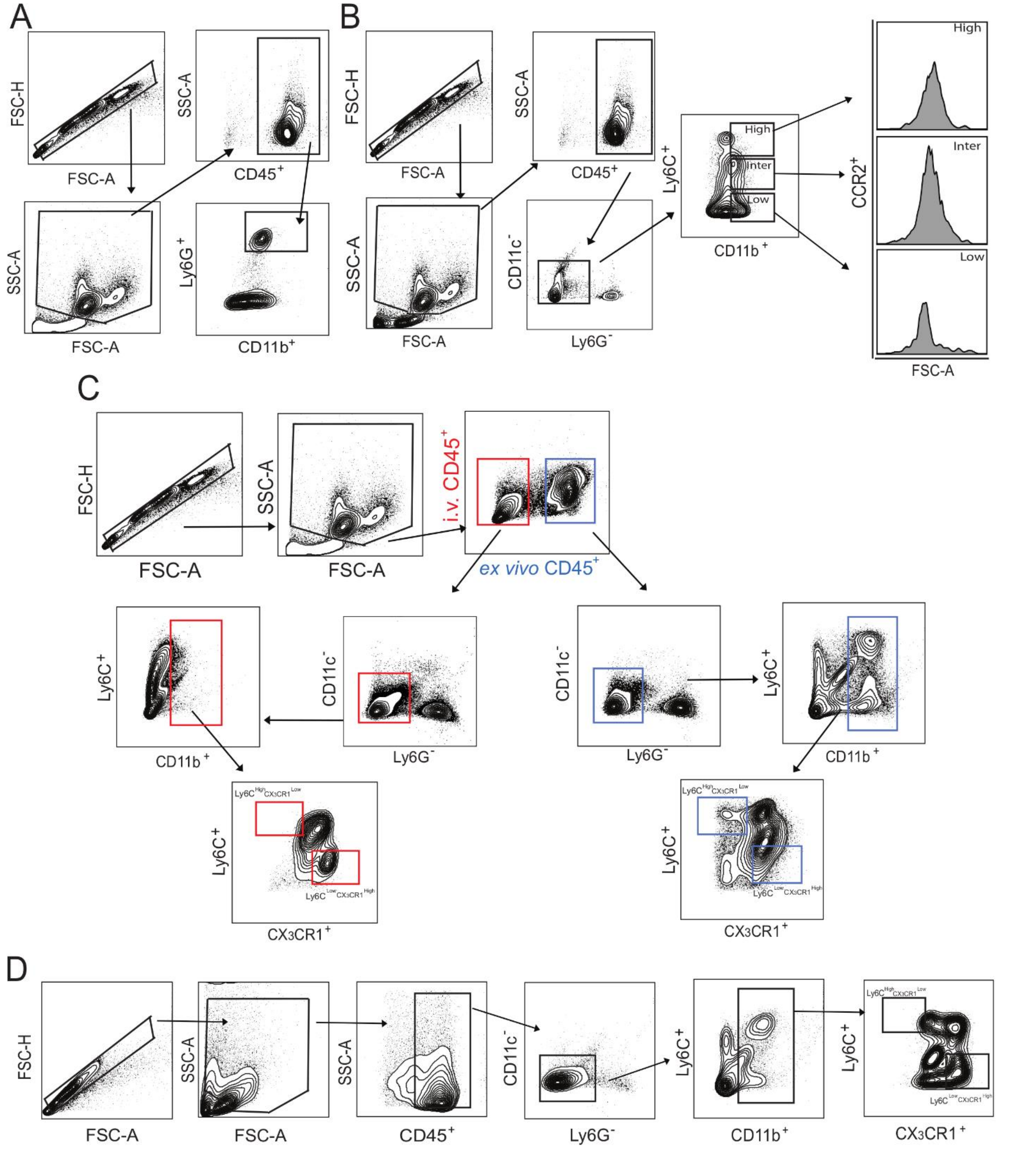

Fig. S1 - Flow cytometric gating strategy. (A) Contour plots show representative flow cytometric gating hierarchy for analysis of FSC-H/ FSCA, SSC-A/ FSC-A followed by CD45^{+} CD11b^{+} Ly6G^{+} neutrophils. **(B)** Contour plots show representative flow cytometric data and gating hierarchy for analysis of FSC-H/ FSCA, SSC-A/ FSC-A followed CD45^{+} CD11c^{-} Ly6G^{-} CD11b^{+} Ly6C^{+} monocyte subsets and subsequent CCR2 mean fluoresce intensity (MFI) calculated for each monocyte subset. **(C)** Contour plots show representative flow cytometric gating hierarchy for analysis of intravascular

leukocyte staining, in which cells were characterized by FSC-H/ FSCA, SSC-A/ FSC-A followed by intravascular or *ex vivo* $CD45^+$ $CD11c^-$ $Ly6G^-$ $CD11b^+$ $Ly6C^+$ $CX3CR1^+$. **(D)** Contour plots show representative flow cytometric gating hierarchy for analysis of FSC-H/ FSCA, SSC-A/ FSC-A followed by $CD45^+$ $CD11c^-$ $Ly6G^-$ $CD11b^+$ $Ly6C^{high}$ $CX3CR1^{low}$ inflammatory (upper gate) or $Ly6C^{low}$ $CX3CR1^{high}$ patrolling monocytes (lower gate).
